# Low Frequency of Asymptomatic and Submicroscopic Plasmodial Infections in Urabá Region in Colombia

**DOI:** 10.1155/2018/8506534

**Published:** 2018-07-02

**Authors:** Carolina Rodríguez Vásquez, Sebastián Barrera Escobar, Alberto Tobón-Castaño

**Affiliations:** ^1^Malaria Group, Faculty of Medicine, Universidad de Antioquia, Medellín, Calle 70 #52-21, Colombia; ^2^Medical Student, Faculty of Medicine, Universidad de Antioquia, Medellín, Colombia

## Abstract

**Background:**

A screening for malaria parasites was conducted with asymptomatic residents in Colombia.

**Methods:**

A descriptive study was carried out in December 2012 in four municipalities of Urabá region in Colombia. A convenience sample of 400 subjects was selected. Participants responded to a survey regarding epidemiological data and blood samples were taken from capillary blood obtained by finger prick for thick smear, rapid diagnostic test (RDT), and polymerase chain reaction (PCR).

**Results:**

399 subjects aged 0.2-98 years were studied (median 22; 221 female (55%)). Episodes of malaria in the last year confirmed by thick film were reported by 47 participants (12%). In 399 samples tested by RDT 4 (1%) were positive (1 with* P. falciparum*, 3 with* P. vivax*), and 3 were confirmed by PCR. In 399 thick blood smears examined 5 (1.3%) were positive (2 with* P. falciparum*, 3 with* P. vivax*), and 3 were confirmed by PCR. In 227 samples, PCR showed 6 (2.6%) positive samples. The parasitaemia was below 1,440 parasites/*μ*L. The best agreement between diagnoses was found between the RDT and thick blood smears (Kappa = 0.75).

**Conclusion:**

Plasmodial afebrile infection was found in 2% of the studied population, by three diagnostic methods, in residents from a low endemic malaria region in Colombia.

## 1. Background

Malaria is an infectious disease caused by various species of Plasmodium, characterized in humans by an acute febrile syndrome with symptoms of general illness such as chills, headache, musculoskeletal pain, and weakness; with low frequency, it presents without fever.

Although the malarial transmission has been declining in prevalence around the world, this protozoan disease remains as an important cause of fatal cases; 429 000 deaths were reported in 2015, most of them (92%) in Africa [1]. Despite this reduction in prevalence, malaria control persists as an important goal in endemic countries; the shift in focus from early diagnosis to active surveillance and treatment of every cases, including those who are asymptomatic, becomes an important issue [2–4].

Asymptomatic infections by* Plasmodium* spp. have not been rigorously defined and generally are referred to as asymptomatic malaria [4–6], which is an imprecise definition. In this topic, the afebrile infection especially has been studied; nevertheless it must be taken into account that this classification leaves out the infections that manifest without fever but with other symptoms. Also, this afebrile infection can show some alteration in hematological parameters and show an increased level of inflammatory markers [7] or contribute to poor outcomes in pregnant women [8].

Asymptomatic infections or asymptomatic carriers can be either microscopic or submicroscopic [5]; the increased use of molecular techniques for detection of parasites with higher sensitivity is reveling the widespread presence of infection below the parasite detection threshold of microscopy and rapid diagnostic tests [9]. Light microscopy misses an average half of all* Plasmodium falciparum* infections in endemic areas [10, 11]; this is explained because light microscopy detects malarial parasites from a burden of 50 per *μ*L of blood [12] in contrast to PCR detection threshold of 0.02 parasites per *μ*L [13].

Some studies indicate that asymptomatic infections are more frequent than symptomatic ones in most malarial endemic settings [6, 14–16]. In a native Amazonian population the prevalence of asymptomatic infection was 4-5 times higher than symptomatic infection [14]; in Solomon islands it was found that only 17.8% of* P. falciparum* and 2.9% of* P. vivax* infected subjects were febrile [17] and in Sao Tome only 3% of the subjects were febrile all harboring* P. falciparum* infection [15]. In contrast to high transmission settings, most of the asymptomatic infections in low transmission settings are submicroscopic [6, 10, 14, 17–19]. In low transmission settings asymptomatic carriers are above 60% of the infected individuals [6, 20]. These conditions are epidemiologically important since they have been related to the persistence of the infection in a determined population [21].

The presence of asymptomatic plasmodial infections in Colombia has been explored in few studies, most of them conducted in Tierralta, Buenaventura, and Tumaco [19, 22–25]. Prevalence of asymptomatic infection detected by PCR was 5.8% in Tumaco and 16.5% in Tierralta, and after a follow-up to 28 days the prevalence of asymptomatic infections by molecular methods was 3.4% in Tumaco and 6.6% in Tierralta [25]. Other observations in Tierralta show a prevalence of asymptomatic carriers around 14.6% [24]. Additionally 1169 subjects enrolled in malaria endemic regions show a frequency of submicroscopic infections in about 12% patients in Buenaventura, 14% in Tierralta, and 4% in Tumaco [19].

In these malarial zones, the frequency of asymptomatic infections by* Plasmodium* spp. has been poorly studied in general population. The aim of this study was to explore the presence of asymptomatic carriers of Plasmodium spp. and describe their epidemiological profile in four municipalities in an endemic region for malaria in Colombia.

## 2. Methods and Materials

### 2.1. Study Area

This descriptive study was conducted in Urabá region, Colombia, where* P. vivax* and* P. falciparum* endemicity is low. Patients were enrolled between 2012 and 2013 in the municipalities of Turbo (8°05′35′′N, 76°43′42′′O), Necoclí (8°25′33′′N, 76°47′02′′O), San Pedro de Urabá (8°16′30′′N, 76°22′37′′O), and Mutatá (7°14′36′′N, 76°26′09′′O).

### 2.2. Study Subjects

A convenience sample of 400 subjects was established. The researchers visited the residents in their home in the rural area. People of any age and sex were invited to participate in the study, who did not show symptoms of the disease in the week before the sample was taken. Demographic aspects as age, sex, place of residence, occupation, residence time in endemic regions for malaria and history of illness, previous malaria episodes, and antimalarial drugs used in the last month were obtained.

### 2.3. Microbiological Tests

From each patient capillary blood was obtained by finger prick for microscopic diagnosis, rapid diagnosis test (RDT), and polymerase chain reaction (PCR). Thick blood smear was prepared according to World Health Organization recommendations [26] and stained with Giemsa, to determine presence of Plasmodium parasites. Two microbiologists with extensive experience in malaria microscopy read the blood slides; the readers were not aware of previous blood smear readings or RDT and PCR results; for the quality control all positive slides were reread by a second microbiologist and any discordance regarding presence or absence of parasites was resolved by a third reading. Parasite densities were calculated by counting the number of asexual parasites per 200 leukocytes, assuming a leukocyte count of 8,000/*μ*L, and the presence of gametocytes was checked. The thick film was considered to be negative, if no parasite had been found in 200 high-power fields, with 1000X magnification.

The RDTs were prepared and read according to manufacturer's instructions (SD Malaria Ag P.f./P.v., Ref. 05FK80, Standard Diagnostics Inc.) by trained medical students who were blinded to blood smear results.

For molecular diagnosis, a sample of blood was taken in Whatman filter paper No. 3, individually stored in a plastic bag and sent to the Laboratory of Malaria Group of the University of Antioquia. The PCR diagnosis is described briefly. The filter paper was added with 0.5% saponin and washed 3 times with 1X PBS and Chelex 100 was added; it was denatured at different temperatures (56°C for 15 minutes and 100°C) and finally the supernatant was recovered after a 15-minute centrifugation at 13000 rpm. Genotyping was done using a PCR protocol developed by Singh et al. [27] by a first amplification reaction with primers rPLU1 and rPLU5 for the fragment of the 18s-rRNA ribosomal subunit of the Plasmodium genus parasites. This PCR product was used for the second reaction (nested PCR) with primers rVIV 1 and rVIV 2 for the identification of P. vivax and rFAL1 and rFAL 2 for the detection of* P. falciparum*.

### 2.4. Statistical Analysis

A descriptive statistical analysis was done with IBM® SPSS® statistics program, 23rd version (licensed to Universidad de Antioquia). The Kolmogorov-Smirnov test was used to assess normality for quantitative variables; the median values were compared with Mann–Whitney U test when variables were not normal. Associations between qualitative variables were analyzed with the Chi-square test. The adopted level of statistical significance was 5% (P value < 0.05).

## 3. Results

From a proposed sample of 400 participants, 399 asymptomatic participants were enrolled in the study: 99 (24.8%) in Turbo, 111 (27.8%) in Necoclí, 35 (8.8%) in Mutatá, and 154 (38.6%) in San Pedro de Urabá. Some demographic aspects are described in Table 1; 55% were woman, with 34% housewives. A previous malaria episode was mentioned by 211 participants (52.8%); 47 (11.8%) mentioned an episode in the last year and 50 (12.5%) participants reported having a history of malaria but did not remember the time when it occurred.

Blood smears and rapid diagnostic tests were performed to all subjects; the PCR was made in 227 subjects. A positive result in any test was considered as an asymptomatic infection and was classified as submicroscopic infection when blood smear was negative and PCR was positive. The blood smear was positive in 5 participants (2 with* P. falciparum*, 3 with* P. vivax*), the rapid diagnostic tests were positive in 4 subjects (1 with* P. falciparum* and 3 with* P. vivax*), and the PCR results were positive in 6 subjects (2 with* P. falciparum* and 4 with* P. vivax*). The frequency of asymptomatic infections detected with any test was 2.0% (8/399) and the frequency of asymptomatic infections by PCR was 2.6% (6/227). The age range of the asymptomatic patients was between 2 and 33 years; 75% were under 15 years.

Between 8 positive subjects, 6 had the three test results, and in this group, the thick blood smear and RDT detected 50% of the infections and PCR detected 100% of the infected subjects (Table 2).

The frequency of 1.3% submicroscopic infections was established between 227 samples analyzed by PCR and microscopy; 3 positive by PCR were negative by microscopy. Between 6 positive samples by PCR, 3 (50.0%) were submicroscopic infections, which were presented in subjects of the mestizo ethnic group, with ages of 8, 12, and 26 years.

The median value of the age in noninfected participants was 22 years (mean 28.5; 95% CI 26.3–30.7); in the infected subjects the median was 10 (mean 13.4; 95% CI 4.5–22.2), without statistical difference (Mann–Whitney U test, P>0.05). Most of the infected subjects were males, students, and of mestizo ethnic group (Table 1).

The median of the time of residency in malarial endemic region was 7. 4 years (3.1–11.6) for the infected subjects against 17.4 years (15.7–17.1) in the noninfected group.

Table 3 presents the positive results.* P. vivax* was the most common agent identified in asymptomatic subjects; no gametocytes were observed.

## 4. Discussion

Identification of asymptomatic carriers of Plasmodium parasites is an important issue for public health due to the epidemiological impact of asymptomatic infections in the perpetuation of the disease in the population and the adverse effects during pregnancy as anemia and low birth weight [8, 28–30] and anemia in children under 5 years [31–33].

Different studies defined asymptomatic malaria as the absence of fever, but clinical manifestations of Plasmodium infection include other findings that usually are not explored and are explained by an increased level of inflammatory markers [7] that could cause other signs and symptoms beside the fever. This can raise the question whether asymptomatic malaria cases are asymptomatic. In this screening, rural residents from four endemic malaria areas without fever or other symptoms were included.

The frequency of asymptomatic infections by* Plasmodium* spp. varies according to the endemicity. In high transmission settings it has been reported that frequencies rise up to 73.7% and 82,0% [34, 35], diagnosed by PCR. In low endemic areas frequencies of 21.7% and 6.4% in the overall population have been reported [14, 17]. Asymptomatic infection between the Plasmodium carrier subjects is above 60% in low transmission settings [6, 16]. In Latin America in a low endemic area from Ecuador a prevalence of 7.5% of asymptomatic infections has been reported [36]. In Colombia recent studies reported asymptomatic infections prevalence of 1% diagnosed by thick blood smear and 9% by PCR in Chocó department [37] and 3,9% of asymptomatic infections were found to be by* P. vivax* in Cauca and 6,7% were found to be by* P. falciparum* in Nariño [23]. In this study a frequency of 2.0% of asymptomatic infections in the Urabá region was found by PCR.

It has been described from previous studies that asymptomatic infections are related to low parasitaemia [19]; a similar finding was revealed in this study with a parasitaemia range between 80 and 1440 parasites/uL. The parasitaemia level is a variable that affects the performance of the rapid diagnostic tests and it is explained by the threshold of parasites detected by RDTs [38, 39]. This study found two negative RDTs in blood samples that corresponded to subjects with positive thick smears, a situation probably explained by the low parasitaemia or by the fact that because of the presence of parasites that lack pfhrp2 gene the enzyme detected by the RDTs is applied [26]. Nevertheless, this genetic deletion has not been described in the parasite population from this region. On the other hand, we found two positive RDTs in individuals that had less than 200 parasites estimated by microscopy.

The importance of asymptomatic patient as a reservoir of the disease rises in two important questions: the duration of infection and the presence of gametocytes [5]. The long standing infections imply more opportunities to get bitten by the mosquito [5] and lead to the gametocytes to appear, as this is a late event in falciparum malaria than in other plasmodia parasites [40]. The presence of gametocytes is a determining factor in transmission of the disease since they infect the mosquito [41]. Some studies have shown that the presence of gametocytes is related to being afebrile [42, 43]; observations in Gambian children found that the risk of gametocytemia in afebrile subjects was 67%, and the presence of low parasitaemia increases the risk fivefold [44]. Similar findings have been described in Nigerian children [45]; interestingly, none of this studies used PCR in their observations. The presence of gametocytes has also been demonstrated in submicroscopic infections [5]; an important issue is that the presence of gametocytes made the infectivity of the mosquitos theatrically possible. In this study there were no gametocytes seen in the infected group; this may be explained by a recent inoculation of sporozoites.

In the natural history of the infection, some of the asymptomatic carriers can become symptomatic. In Brazil it was found that 10.7% of asymptomatic individuals became symptomatic in a period of two months [46]; other observations from children between one to five years and a follow-up of 33 days showed that 55.9% became febrile and showed some spikes in the parasite density [47]. Observations in Tanzania also confirm these findings showing that 7.9% of asymptomatic infections will be accompanied by fever in a variable time [48] and another study has found that in highly endemic areas for* P. falciparum* the infection had a median duration of 194 days (95% CI: 191-196) [49].

A study conducted in Uganda showed that 39% of children between 6 months and 5 years with subpatent infection became symptomatic in 20 weeks in comparison to 82% of children with patent infection [50] concluding that microscopic parasitaemia is more likely to become symptomatic than submicroscopic parasitaemia.

In this study, asymptomatic infection was found in 8 subjects; however, because there was no follow-up, it raises the question of whether this parasitaemia is detected in the last moment of the incubation period; given the short time between the completion of the prepatent period and the end of the incubation period (1 day for* P. falciparum* and 4 days for* P. vivax*) this is likely an infrequent occurrence [6].

An important conclusion of these findings is that the active search of cases diagnosed by light microscopy is insufficient to find all the asymptomatic carriers; the lower threshold of parasite detection in the field by microscopy has been estimated in 50-100 parasites/*μ*L [12] similar to those described for RDT, ~100 parasites per *μ*l of blood in approximately 5 *μ*l of whole blood [12], in contrast to the lower threshold for PCR (a threshold of detection of 0.02 parasites/*μ*L for most sensitive methods) [13] that theoretically can detect 1 gene copy per reaction or 1 parasite in the blood sample [16]. Observations in Tierralta, another endemic region in Colombia, showed that the PCR detects 50% more infected patients than light microscopy [24]; these findings are according to ours.

It is important to keep in mind that, in the areas where parasite prevalence established by light microscopy is less than 10%, the proportion of submicroscopic infections is estimated up to 70-80% and these infections would be responsible for more than 50% of human to mosquito infection [51]. Based on this data we can conclude that active screening with light microscopy is insufficient for screening in low endemic regions to find these subjects.

An interesting observation is that we found that* P. vivax* was the most common agent implicated in asymptomatic malaria; this in contrast with other results of asymptomatic malaria in Latin America. In Brazil,* P. falciparum* was diagnosed more frequently (37.5%) than* P. vivax* (18.5%) [46] and other results from Brazil show that the prevalence of infection by species was not statically different although* P. vivax* infection was slightly higher [14]; maybe this can be explained by the fact that* P. vivax* is the most common Plasmodium parasite in Brazil. Observations in Colombia show that asymptomatic infections are more prevalent in* P. vivax* than in* P. falciparum* infections; in Tierralta 64.5% of asymptomatic infections were caused by* P. vivax*, 29% by* P. falciparum*, and 6,5% by mixed infection of these species; asymptomatic cases were more common in men than in women [24]. In contrast to these findings, in Urabá region,* P. falciparum* was present in 56% of asymptomatic plasmodial infections in women diagnosed by qPCR,* P. vivax* in 41,5%, and mixed infection in 2.5% [22]. Another study compares the prevalence of asymptomatic infection in Tierralta, Buenaventura, and Tumaco; the prevalence of* P. vivax* was 93%, 66%, and 50%, respectively [19]. Another finding similar to those shows that* P. vivax* was more prevalent in Tierralta but* P. falciparum* was more prevalent in Tumaco [25]. A cross-sectional study with a follow-up of four years shows that* P. vivax* was more prevalent in Tierralta and* P. falciparum* was more common in Buenaventura, and despite malarial decreasing trend in the region, the proportion of* P. vivax* did not change significantly between 2011 and 2013 [23]. This distribution of asymptomatic infections by species could be explained by the different distribution of the parasite in Colombia, which can also influence our results.

It has been described that adults are more likely to develop submicroscopic infections than children [51, 52]. Partial immunity can explain the differences between the age in symptomatic or asymptomatic subjects. The immunity against the disease is acquired after parasite infection; observations in areas with moderate to high malaria transmission show a decrease in symptomatic illness near the adolescence [53]. Two types of partially immune reaction have been described in malaria, first a protection against clinical disease that develops faster and is exposure dependent and an antiparasitic response that develops later and has a longer duration (20 years or more) [53]; consistent with this, adults are more likely than children to develop submicroscopic infections [51, 52]; this cannot be evidenced in this study, possibly due to the low frequency of submicroscopic infections detected; on the contrary, we found that the highest frequency of asymptomatic (75%) and submicroscopic infections (66.7%) occurred in children under 15 years of age. This situation requires studies in low endemic areas to improve their understanding.

In areas of low malaria endemicity, several studies show that subclinical infections with very low parasite loads are quite common [16], and not only antidisease immunity but also some degree of antiparasite immunity is acquired in rural villages. In a Colombian study about the prevalence of asymptomatic infections, most of the subjects with asymptomatic infection were under 30 years, with the highest prevalence between 10 and 20 years [25]; we found a majority of asymptomatic infections in children between 2 and 12 years (6/8; 75%). This is a possible protection against clinical disease acquired by recent infections or because the infected subjects have more antecedents of malaria episodes, however without statistical difference (P=0.056) maybe due to the sample size. More studies with larger samples are required to confirm these findings.

## 5. Conclusion

We found the presence of asymptomatic plasmodial infections in a malarial region of low endemicity in Colombia, with an important component of submicroscopic malaria, which showed that light microscopy is an insufficient technique for active patient screening and to eradicate malaria. Asymptomatic infections were found especially in children, which may indicate a high exposure to the parasite and demand surveillance through more sensitive diagnostic methods in this risk group and in the general population.

## Figures and Tables

**Table 1:** Sociodemographic characterization.

	**Subjects**	**Total** n=399 **n (**%**)**	**Asymptomatic infection** n=8 **n (**%**)**
**Sex** **∗**	Male	178 (44.6)	7 (87.5)
	Female	221 (55.4)	1 (12.5)

**Ethnicity**	Afro-Americans	46 (11.5)	1 (12.5)
	Indigenous	11 (2.8)	-
	Mestizo	341 (85.5)	7 (87.5)
	Caucasian	1 (0.3)	-

	Housewife	135 (33.8)	1 (12.5)
	Student	121 (30.3)	5 (62.5)
**Occupation**	Farmer	56 (14)	1 (12.5)
	Merchant	5 (1.3)	-
	Office worker	1 (0.3)	-
	Arts and crafts	13 (3.3)	-
	Others	8 (2)	1 (12.5)

**Previous episode of malaria**	Less than one month	10 (2.5)	1 (12.5)
	One to six months	22 (5.5)	1 (12.5)
	Seven to twelve months	15 (3.8)	1 (12.5)
	> 1 year *∗∗*	114 (28.6)	-
	Unknown date	50 (12.5)	-

**Last Plasmodium species diagnosed **(any time)	*P. Vivax*	93 (23.3)	3 (37.5)
	*P. Falciparum*	16 (4.1)	-
	Mixed	10 (2.5)	-
	Unknown	92 (23.1)	-

*∗*Chi-square test; P=0.025 (Fisher test); *∗∗*Chi-square test; P=0.056 (Fisher test).

**Table 2:** Thick blood smear, RDT, and PCR positive results per municipality and species of *Plasmodium*.

**Sample per site / Test**	**Thick blood Smear**	**RDT**	**PCR**	**Any test positive** **∗**
	(n=399)	(n=399)	(n=227)	(n=399)
Turbo (n= 99; 24.8%)	1 (1.0%)	1 (1.0%)	3 (3.0%)	3 (3.0%)
Necoclí (n=111; 27.8%)	1 (0.9%)	1 (0.9%)	0 - - - -	1 (0.9%)
San Pedro (n=154; 38.6%)	3 (2.0%)	2 (1.3%)	2 (1.3%)	3 (2.0%)
Mutatá (n=35; 8.8%)	0 - - - - - -	0 - - - - -	1 (2.9%)	1 (2.9%)
**Total (n=399; 100%)**	**5 (1.3)**	**4 (1.0%)**	**6 (2.2%)**	**8 (2.0%)**

**Specie / Test**				

*Plasmodium falciparum*	2 (0.5%)	1 (0.3%)	2 (0.7%)	3 (0.8%)
*Plasmodium vivax*	3 (0.7%)	3 (0.7%)	4 (1.4%)	5 (1.3%)
**Total (n=399; 100%)**	**5 (1.3%)**	**4 (1.0%)**	**6 (2.2%)**	**8 (2.0%)**

*∗*Positive result for a sample with at least one of the tests performed.

**Table 3:** Asymptomatic infections in Urabá region diagnosed by microscopy and molecular methods.

**Patient**	**Thick Smear**	**RDT**	**PCR**	**Parasitaemia**
				**P/uL**
**1**	*P. vivax*	*P. vivax*	Not available	160
**2**	*P. vivax*	*P. vivax*	*P. vivax*	1440
**3**	*P. vivax*	*P. vivax*	*P. vivax*	160
**4**	*P. falciparum*	Negative	*P. falciparum*	140
**5**	*P. falciparum*	Negative	Not available	80
**6**	Negative	*P. falciparum*	*P. falciparum*	-
**7**	Negative	Negative	*P. vivax*	-
**8**	Negative	Negative	*P. vivax*	-

## References

[1] World Health Organization (2016). *World Malaria Report*.

[2] Sturrock H. J. W., Hsiang M. S., Cohen J. M. (2013). Targeting Asymptomatic Malaria Infections: Active Surveillance in Control and Elimination. *PLoS Medicine*.

[3] Mendis K., Rietveld A., Warsame M., Bosman A., Greenwood B., Wernsdorfer W. H. (2009). From malaria control to eradication: the WHO perspective. *Tropical Medicine & International Health*.

[4] Smith Gueye C., Sanders K. C., Galappaththy G. N. (2013). Active case detection for malaria elimination: A survey among Asia Pacific countries. *Malaria Journal*.

[5] Lin J. T., Saunders D. L., Meshnick S. R. (2014). The role of submicroscopic parasitemia in malaria transmission: what is the evidence?. *Trends in Parasitology*.

[6] Lindblade K. A., Steinhardt L., Samuels A., Kachur S. P., Slutsker L. (2013). The silent threat: Asymptomatic parasitemia and malaria transmission. *Expert Review of Anti-infective Therapy*.

[7] de Mast Q., Brouwers J., Syafruddin D. (2015). Is asymptomatic malaria really asymptomatic? Hematological, vascular and inflammatory effects of asymptomatic malaria parasitemia. *Infection*.

[8] Cottrell G., Moussiliou A., Luty A. J. F. (2015). Submicroscopic plasmodium falciparum infections are associated with maternal anemia, premature births, and low birth weight. *Clinical Infectious Diseases*.

[9] Snounou G., Brown K. N., Fonseca L. (1993). The importance of sensitive detection of malaria parasites in the human and insect hosts in epidemiological studies, as shown by the analysis of field samples from guinea bissau. *Transactions of the Royal Society of Tropical Medicine and Hygiene*.

[10] Okell L. C., Ghani A. C., Lyons E., Drakeley C. J. (2009). Submicroscopic infection in *Plasmodium falciparum*-endemic populations: a systematic review and meta-analysis. *The Journal of Infectious Diseases*.

[11] Rek J., Katrak S., Obasi H. (2016). Characterizing microscopic and submicroscopic malaria parasitaemia at three sites with varied transmission intensity in Uganda. *Malaria Journal*.

[12] Wongsrichanalai C., Barcus M. J., Muth S., Sutamihardja A., Wernsdorfer W. H. (2007). A review of malaria diagnostic tools: microscopy and rapid diagnostic test (RDT). *The American Journal of Tropical Medicine and Hygiene*.

[13] Mahajan B., Zheng H., Pham P. T. (2012). Polymerase chain reaction-based tests for pan-species and species-specific detection of human Plasmodium parasites. *Transfusion*.

[14] Alves F. P., Durlacher R. R., Menezes M. J., Krieger H., Pereira da Silva L. H., Camargo E. P. (2002). High prevalence of asymptomatic Plasmodium vivax and *Plasmodium falciparum* infections in native Amazonian populations. *The American Journal of Tropical Medicine and Hygiene*.

[15] Pinto J., Sousa C. A., Gil V. (2000). Malaria in Sao Tome and Principe: Parasite prevalences and vector densities. *Acta Tropica*.

[16] Bousema T., Okell L., Felger I., Drakeley C. (2014). Asymptomatic malaria infections: Detectability, transmissibility and public health relevance. *Nature Reviews Microbiology*.

[17] Harris I., Sharrock W. W., Bain L. M. (2010). A large proportion of asymptomatic Plasmodium infections with low and sub-microscopic parasite densities in the low transmission setting of Temotu Province, Solomon Islands: Challenges for malaria diagnostics in an elimination setting. *Malaria Journal*.

[18] Steenkeste N., Rogers W. O., Okell L. (2010). Sub-microscopic malaria cases and mixed malaria infection in a remote area of high malaria endemicity in Rattanakiri province, Cambodia: Implication for malaria elimination. *Malaria Journal*.

[19] Vallejo A. F., Chaparro P. E., Benavides Y. (2015). High prevalence of sub-microscopic infections in Colombia. *Malaria Journal*.

[20] Greenwood B. M. (1987). Asymptomatic malaria infections—do they matter?. *Parasitology Today*.

[21] Roshanravan B., Kari E., Gilman R. H. (2003). Endemic malaria in the Peruvian Amazon region of Iquitos. *The American Journal of Tropical Medicine and Hygiene*.

[22] Carmona-Fonseca J., Agudelo O. M., Arango E. M. (2017). Asymptomatic plasmodial infection in Colombian pregnant women. *Acta Tropica*.

[23] Vásquez-Jiménez J. M., Arévalo-Herrera M., Henao-Giraldo J. (2016). Consistent prevalence of asymptomatic infections in malaria endemic populations in Colombia over time. *Malaria Journal*.

[24] Cucunubá Z. M., Guerra Á. P., Rahirant S. J., Rivera J. A., Cortés L. J., Nicholls R. S. (2008). Asymptomatic Plasmodium spp. infection in Tierralta, Colombia. *Memórias do Instituto Oswaldo Cruz*.

[25] Cucunubá Z. M., Guerra Á. P., Rivera J. A., Nicholls R. S. (2013). Comparison of asymptomatic plasmodium spp. infection in two malaria-endemic colombian locations. *Transactions of the Royal Society of Tropical Medicine and Hygiene*.

[26] Storey J., Organización Mundial de la Salud (2009). *Bases del Diagnóstico Microscópico del Paludismo*.

[27] Singh B., Bobogare A., Cox-Singh J., Snounou G., Abdullah M. S., Rahman H. A. (1999). A genus- and species-specific nested polymerase chain reaction malaria detection assay for epidemiologic studies. *The American Journal of Tropical Medicine and Hygiene*.

[28] Saute F., Menendez C., Mayor A. (2002). Malaria in pregnancy in rural Mozambique: the role of parity, submicroscopic and multiple *Plasmodium falciparum* infections. *Tropical Medicine & International Health*.

[29] Arango E., Maestre A., Carmona-Fonseca J. (2010). Effect of submicroscopic or polyclonal Plasmodium falciparum infection on mother and gestation product. *Revista Brasileira de Epidemiologia*.

[30] Nega D., Dana D., Tefera T., Eshetu T. (2015). Anemia associated with asymptomatic malaria among pregnant women in the rural surroundings of Arba Minch Town, South Ethiopia. *BMC Research Notes*.

[31] Verhoef H., West C. E., Kraaijenhagen R. (2002). Malarial anemia leads to adequately increased erythropoiesis in asymptomatic Kenyan children. *Blood*.

[32] Maketa V., Mavoko H., da Luz R. (2015). The relationship between *Plasmodium* infection, anaemia and nutritional status in asymptomatic children aged under five years living in stable transmission zones in Kinshasa, Democratic Republic of Congo. *Malaria Journal*.

[33] Akiyama T., Pongvongsa T., Phrommala S. (2016). Asymptomatic malaria, growth status, and anaemia among children in Lao People’s Democratic Republic: a cross-sectional study. *Malaria Journal*.

[34] Geiger C., Agustar H. K., Compaoré G. (2013). Declining malaria parasite prevalence and trends of asymptomatic parasitaemia in a seasonal transmission setting in north-western Burkina Faso between 2000 and 2009-2012. *Malaria Journal*.

[35] Owusu-Agyei S., Smith T., Beck H.-P., Amenga-Etego L., Felger I. (2002). Molecular epidemiology of Plasmodium falciparum infections among asymptomatic inhabitants of a holoendemic malarious area in northern Ghana. *Tropical Medicine & International Health*.

[36] Sáenz F. E., Arévalo-Cortés A., Valenzuela G. (2017). Malaria epidemiology in low-endemicity areas of the northern coast of Ecuador: high prevalence of asymptomatic infections. *Malaria Journal*.

[37] Molina Gómez K., Caicedo M. A., Gaitán A. (2017). Characterizing the malaria rural-to-urban transmission interface: The importance of reactive case detection. *PLOS Neglected Tropical Diseases*.

[38] Murray C. K., Gasser R. A., Magill A. J., Miller R. S. (2008). Update on rapid diagnostic testing for malaria. *Clinical Microbiology Reviews*.

[39] World Health Organization (2012). *Malaria Rapid Diagnostic Test Performance*.

[40] Bousema T., Drakeley C. (2011). Epidemiology and infectivity of *Plasmodium falciparum* and *Plasmodium vivax* gametocytes in relation to malaria control and elimination. *Clinical Microbiology Reviews*.

[41] White N. J., Pukrittayakamee S., Hien T. T. (2013). Malaria. *The Lancet*.

[42] Price R., Nosten F., Simpson J. A. (1999). Risk factors for gametocyte carriage in uncomplicated falciparum malaria. *The American Journal of Tropical Medicine and Hygiene*.

[43] Sumari D., Mwingira F., Selemani M., Mugasa J., Mugittu K., Gwakisa P. (2017). Malaria prevalence in asymptomatic and symptomatic children in Kiwangwa, Bagamoyo district, Tanzania. *Malaria Journal*.

[44] Von Seidlein L., Drakeley C., Greenwood B., Walraven G., Targett G. (2001). Risk factors for gametocyte carriage in Gambian children. *The American Journal of Tropical Medicine and Hygiene*.

[45] Sowunmi A., Fateye B. A., Adedeji A. A., Fehintola F. A., Happi T. C. (2004). Risk factors for gametocyte carriage in uncomplicated falciparum malaria in children. *Parasitology*.

[46] Da Silva-Nunes M., Ferreira M. U. (2007). Clinical spectrum of uncomplicated malaria in semi-immune Amazonians: beyond the ‘symptomatic’ vs ‘asymptomatic’ dichotomy. *Memórias do Instituto Oswaldo Cruz*.

[47] Magesa S. M., Mdira K. Y., Babiker H. A. (2002). Diversity of Plasmodium falciparum clones infecting children living in a holoendemic area in north-eastern Tanzania. *Acta Tropica*.

[48] Bereczky S., Montgomery S. M., Troye-Blomberg M., Rooth I., Shaw M., Färnert A. (2004). Elevated anti-malarial IgE in asymptomatic individuals is associated with reduced risk for subsequent clinical malaria. *International Journal for Parasitology*.

[49] Felger I., Maire M., Bretscher M. T. (2012). The Dynamics of Natural Plasmodium falciparum Infections. *PLoS ONE*.

[50] Nsobya S. L., Parikh S., Kironde F. (2004). Molecular evaluation of the natural history of asymptomatic parasitemia in Ugandan children. *The Journal of Infectious Diseases*.

[51] Okell L. C., Bousema T., Griffin J. T., Ouedraogo A. L., Ghani A. C., Drakeley C. J. (2012). Factors determining the occurrence of submicroscopic malaria infections and their relevance for control. *Nature Communications*.

[52] Mosha J. F., Sturrock H. J., Greenhouse B. (2013). Epidemiology of subpatent Plasmodium falciparum infection: Implications for detection of hotspots with imperfect diagnostics. *Malaria Journal*.

[53] Filipe J. A., Riley E. M., Drakeley C. J., Sutherland C. J., Ghani A. C. (2007). Determination of the processes driving the acquisition of immunity to malaria using a mathematical transmission model. *PLoS Computational Biology*.

